# Internet-Related Addictions: From Measurements to Interventions

**DOI:** 10.3390/ijerph17072539

**Published:** 2020-04-08

**Authors:** Phoenix K. H. Mo, Juliet Honglei Chen, Joseph T. F. Lau, Anise M. S. Wu

**Affiliations:** 1 Center for Health Behaviours Research, School of Public Health and Primary Care, The Chinese University of Hong Kong, Hong Kong, China; phoenix.mo@cuhk.edu.hk (P.K.H.M.); jlau@cuhk.edu.hk (J.T.F.L.); 2 Department of Psychology, Faculty of Social Sciences, University of Macau, Macao, China; JulietHChen@outlook.com; 3 Center for Cognitive and Brain Sciences, University of Macau, Macao, China

Ever since the invention of the World Wide Web in 1989 [1], the Internet has dramatically reshaped all walks of life. It has also expanded the spectrum of Internet-related disorders in the fifth edition of the Diagnostic and Statistical Manual of Mental Disorders (DSM-5) and the eleventh revision of the International Classification of Disease (ICD-11) [2,3]. Research on Internet-related addictions can be dated back to 1996 when problems associated with excessive Internet use in general, such as Internet Addiction (IA), were studied [4]; IA has been found to cause various physical problems and psychopathology among adolescents and adults [5]. There are different types of Internet-related addiction [6,7], such as Internet gaming disorder (IGD), problematic mobile phone use (PMPU), social networking addiction (SNA), and Internet gambling. These subtypes have common (e.g., heightened impulsivity) and specific (e.g., maladaptive expectancies) characteristics [8]. This Special Issue has gathered 15 papers that have looked into important topics of research on various types of Internet-related addiction.

First, as the cornerstone of studying Internet-related addictions, it is essential to improve the assessment accuracy for a specific addiction. Ko et al. [9] adapted a gaming version of the commonly used Chen Internet Addiction Scale, the CIAS-G, to assess the *DSM-5* IGD based upon data of 69 clinically diagnosed IGD cases, 69 regular Internet gamers, and 69 healthy Internet gamers in Taiwan. The screening (≥68), diagnostic (≥72), and prevalence-estimate (≥76) cutoff points were proposed to identify probable IGD for different clinical and epidemiological utility. Future studies may further validate this tool and compare it with existing tools, especially those based on the ICD-11 criteria. While many studies investigated the cognitions of Internet-related addictions and associated impacts [10], there is a dearth of studies validating measurement tools to assess such cognitions in a systematic way. Yu et al. [11] validated and further revised the Internet Gaming Cognition Scale, which was originally based on a systematic review of cognitive factors of IGD, among 755 Chinese junior high school students. The authors identified three types of maladaptive Internet gaming cognitions, including perceived rewards of Internet gaming, perceived urges for playing Internet games, and perceived unwillingness to stop playing without completion of gaming tasks. These maladaptive cognitions were found to be significantly associated with heightened impulsivity, impaired self-control, increased gaming time, and higher risk for IGD. This validation study strengthens research on relationships between cognitions and IGD. Relatedly, Chia et al. [12] conducted a scoping review of cognitive bias in IA and IGD. Of the six identified studies, five demonstrated existence of cognitive biases among individuals with IA/IGD; one study demonstrated effectiveness of bias modification for IGD. Cognitive bias and bias modifications related to IA/IGD are important research topics that deserve more research and clinical attention.

Second, five studies looked at a variety of health-related harms associated with different types of problematic Internet use. Machimbarrena et al. [13] classified 12,285 Spanish adolescent Internet users into four groups, non-problematic users (57.9%), mood regulators (18.7%), problematic Internet users (18.5%), and severe problematic Internet users (4.9%). They found that the two groups of problematic Internet users were more likely to report lower health-related quality of life. Mo et al. [14] reported a significant association between SNA and smoking, which was mediated by depressive symptomatology and low social support among Chinese junior secondary school students (*N* = 5182). Li, Yang et al. [15] reported high prevalence of non-suicidal self-injury (NSSI) of 32.1% among Chinese junior and high school students (*N* = 22,628), which was significantly associated with PMPU. Furthermore, PMPU interacted significantly with low health literacy to determine the risk of NSSI. Tang et al. [16] found that IA was significantly associated with higher level of depression and subsequently more support for radical actions during a massive socio-political movement among university students in Hong Kong (*N* = 290). It is probably the first report of the potential impact of IA on behaviors related to social unrest. Hui et al. [17] applied the self-determination theory to explore the potential effects of the satisfaction and dissatisfaction of basic psychological needs (i.e., needs for autonomy, competence, and relatedness) on eudaimonic well-being, as indicated by flourishing, and the mediating role of IGD among 1200 Chinese young adults. IGD was found to mediate the negative effect of need dissatisfaction for relatedness on flourishing. These findings reveal an important message that various types of problematic Internet use are potentially harmful, although they have not been included into the DSM-5 or the ICD-11. Thus, interventions for such ‘non-disease’ addictive behaviors are still warranted.

Design of interventions requires knowledge about related risk factors of Internet-related addictions. Given the social connection function of the Internet, several studies of this Special Issue highlight the importance of interpersonal factors of IGD/IA. As aforementioned, need dissatisfaction for relatedness was identified as a risk factor of IGD for Chinese young adults by Hui et al. [17]. Yang et al. [18] found four types of interpersonal stressors that may exacerbate adolescent IGD, which include parental psychological control, physical/verbal abuse by parents, verbal abuse by teachers, and peer/online bullying (*N* = 2666). In addition to the neurotic personality trait, Chang et al. [19] also revealed that the wish to develop new interpersonal relationships online was a potential antecedent of IA among young adults in Taiwan (*N* = 223), via the mediating effect of anxiety during online interpersonal interactions. On the other hand, Wartberg and Lindenberg [20] conducted a one-year follow-up study on a group of 272 German adolescents at risk for problematic Internet use (PIU), and showed the salient adverse effect of maladaptive emotion regulation strategies, among other risk psychosocial predictors including lower self-efficacy, higher anxiety towards school and school-related performance, higher social-interaction anxiety, higher procrastination, and higher depression. The reports of this Special Issue also remind us about the importance of protective factors of IGD. Yang et al. [20] showed that supportive and positive relationships with parents and peers were protective against adolescent IGD (*N* = 2666). Based on the Interaction of Person-Affect-Cognition-Execution model, Dang et al. [21] also found the protective role of emotional intelligence and coping flexibility against IGD through decreasing depressive symptoms among 282 Chinese university students in their one-year longitudinal study.

In the absence of discussion about interventions, this Special Issue would be an incomplete one. Li, Chau et al. [22] evaluated a new parent-based gaming disorder intervention program, the Game Over Intervention (GOI), among Hong Kong upper primary school students (*N* = 163 intervention and 199 control). Based on the frameworks of ecological systems theory and self-determination theory, this program manifested efficacies in ameliorating some gaming-related problems (e.g., reduction in children’s gaming time, less exposure to violent video games, and fewer symptoms of gaming disorder) over a three-month follow-up period. Alrobai et al. [23] proposed intervention protocols to combat digital addiction (DA) with a systematic and participatory approach. Based on two observational studies and one case study conducted in the U.K., they developed a reference model for designing interactive online platforms to intervene DA with peer groups, as well as a process model (i.e., COPE.er) for building a customizable online environment for different disease groups. Echoed with the aforementioned findings on interpersonal risk/protective factors [17,18,19], both interventions involved interpersonal elements.

Last but not least, researchers should think about future research directions. Lawn et al. [24] conducted a gap analysis commissioned by the Office of Responsible Gambling in New South Wales, Australia, and reviewed 116 papers related to emerging technologies or new trends of gambling. They discovered Internet gambling as the main area of focus, and identified specific gaps regarding methods (e.g., more longitudinal studies), knowledge (e.g., potential harm arising from technological development), and public health/practical knowledge (e.g., responsible gambling policies or initiatives).

This Special Issue has thus expanded research areas of Internet-related addictions. It has confirmed findings of previous studies, e.g., [25,26], and brought new insights to this important subject area. The reports highlight the importance of valid measurement tools, epidemiological investigations of harms and potential determinants, and attempts to develop evidence-based interventions related to various types of Internet-related addiction. Its richness reminds us that although IGD and gambling disorder are the only two disorders included by the DSM-5 and the ICD-11, other forms of Internet-related addictions are important and consequential. Future research may look at their commonalities and differences from measurements to interventions.
